# Strategic investments in non-communicable diseases (NCD) research in Africa: the GSK Africa NCD Open Lab

**DOI:** 10.5830/CVJA-2015-042

**Published:** 2015

**Authors:** Matthew D Hall, Ann M Dufton, Roy M Katso, Pauline M Williams, Sally A Gatsi, Michael E Strange

**Affiliations:** GSK, Medicines Research Centre, Stevenage, Hertfordshire, United Kingdom; GSK, Medicines Research Centre, Stevenage, Hertfordshire, United Kingdom; GSK, Medicines Research Centre, Stevenage, Hertfordshire, United Kingdom; GSK, Medicines Research Centre, Stevenage, Hertfordshire, United Kingdom; GSK, Brentford, Middlesex, United Kingdom; GSK, Brentford, Middlesex, United Kingdom

**Keywords:** non-communicable diseases, Africa, open innovation, collaboration, training, capacity building

## Abstract

In March 2014, GSK announced a number of new strategic investments in Africa. One of these included investment of up to 25 million Pounds Sterling (£25 million) to create the world’s first R&D Open Lab to increase understanding of non-communicable diseases (NCDs) in Africa. The vision is to create a new global R&D effort with GSK working in partnership with major funders, academic centres and governments to share expertise and resources to conduct high-quality research. The Africa NCD Open Lab will see GSK scientists collaborate with scientific research centres across Africa. An independent advisory board of leading scientists and clinicians will provide input to develop the strategy and selection of NCD research projects within a dynamic and networked open-innovation environment. It is hoped that these research projects will inform prevention and treatment strategies in the future and will enable researchers across academia and industry to discover and develop new medicines to address the specific needs of African patients.

## Abstract

In 2012, the World Health Assembly endorsed an important new health goal (the ‘25 by 25’ goal) to reduce avoidable mortality from non-communicable diseases (NCDs) by 25% by 2025.^1^ Furthermore, the 66th World Health Assembly, United Nations (UN) member states adopted a resolution on NCDs that reinforces commitments made in 2011’s UN Declaration on NCDs and signals consensus on the three pillars of the global NCD architecture – action, accountability and coordination.^2^ This alignment of the UN’s World Health Assembly, the World Health Organisation, and other entities are recognised as positive steps for combating NCDs.

NCDs account for 63% of all deaths globally, with 80% of the global burden occurring in low- and middle-income countries.^3^ While NCDs are currently the leading cause of death in all regions except Africa, current projections indicate that by 2020, the largest increases in NCD deaths will occur in Africa. Furthermore, by 2030, the number of deaths from NCDs in Africa is projected to exceed the combined deaths from communicable, nutritional, maternal and perinatal deaths as the most common causes of death, if current trends continue.^3^

Currently, NCDs are increasing in Africa. Mbanya and colleagues found that approximately 12 million people were living with diabetes in Africa in 2010; this is projected to increase to approximately 24 million by 2030.^4^ Other contributors to the increase in diabetes may also include anti-retroviral treatment (ART) for HIV/AIDS. ART has been shown to increase the risk of cardiometabolic dysregulation; this dysregulation is associated with obesity and increased insulin resistance.^5^ Therefore increase in incidences of NCDs will result from the aforementioned factors, including the increased lifespan of people living with infectious diseases.

GSK understands that there is much more to be done to appreciate and address the burden of NCDs, with a particular focus on Africa. As a science-led global healthcare company, GSK is committed to harnessing its scientific expertise, partnerships and global reach to develop and make products for people who need them, wherever they live. Research and development areas include HIV, vaccines, and diseases of the developing world. GSK has pioneered a number of innovative new models to help stimulate innovation: working in partnership with others, and opening up access to its expertise, facilities and intellectual property.^6^

For example, the Tres Cantos Open Lab Foundation was established by GSK in 2010 and offers a unique approach to discovering novel, safe, appropriate and affordable medicines for diseases of the developing world with industry, academia, non-governmental organisations and governments working together. The Tres Cantos Open Lab is the world’s first open laboratory for diseases of the developing world and provides an opportunity for scientists from around the world to partner with GSK scientists, using GSK’s facilities and expertise, to test their own ideas and design appropriate projects at the very early stages of drug discovery.^7^

Building on this and other initiatives, GSK’s chief executive officer, Sir Andrew Witty, announced new strategic investments in sub-Saharan Africa at the 5th European Union–Africa Business Forum in Brussels in March 2014. These investments also included the Africa NCD Open Lab, which was designed to address pressing health needs and contribute to long-term business growth. Over the next five years, GSK will make targeted investments of up to £130 million in Africa, which will contribute to the development of home-grown capabilities and skills in Africa.^8^ The long-term goal is to equip Africa to discover, develop and produce the medicines required for African patients.

The projected increase in NCDs across developing countries offers an opportunity for GSK to participate alongside global players to help improve the collective understanding of the specific variations of disease in low-resource settings. Building on the success of GSK’s Tres Cantos Open Lab, the company’s vision is to create a new global R&D effort, the Africa NCD Open Lab, with GSK working in partnership with African researchers to conduct NCD-related research in Africa. The results of this research will be published in order to disseminate relevant insights to the wider community. To do this, GSK will fully engage with the scientific and clinical research communities in Africa to bring on-the-ground expertise and experience to address the problem.

## GSK working in global collaboration

GSK recognises that by working in partnership with non-governmental organisations, governments, academic institutions and other companies, it can achieve more for patients than it can alone. It currently has research collaborations with more than 3 000 external organisations, including other companies, academic institutions and research charities.^9^ For example, a five-year strategic partnership with Save the Children aims to help save one million children’s lives.^10^ The partnership will combine expertise, resources, and capabilities and bring much-needed medicines and vaccines to some of the world’s poorest children, train thousands of healthcare workers, and develop medicines to address diseases in these paediatric populations. As part of the Save the Children partnership, GSK’s Maternal and Neonatal R&D Unit is developing a gel form of a GSK antiseptic product used in mouthwash to help prevent sepsis in newborn babies.

## Vision for Africa NCD Open Lab

Improving healthcare infrastructure and access to care is a key element of addressing NCDs in Africa. Before discovering and developing new medicines specifically for African patients, more needs to be done to understand the burden of these diseases. In addition, there are significant gaps in our knowledge about the diversity of the causes of NCDs in Africa, their presentation, and the responses to medicines (possibly driven by genetic variation, environmental influence or behavioural factors).

To address the knowledge gaps, the research will focus on better understanding the unique aspects (e.g. genotypic, phenotypic, cultural and environmental context) of NCDs in the African setting through translational research that will integrate basic laboratory-based, clinical and population-based research. New research is required to better understand how these diseases develop, how they present, and how patients can best be treated in the African context.

The Africa NCD Open Lab will be centred at GSK’s R&D hub in Stevenage, UK, which together with the multiple partnerships, including with local African research institutes, will provide a world-class, dynamic and highly networked R&D environment that will deliver high-quality and impactful research outputs. This environment will provide a unique opportunity to strengthen African research capability and train a new generation of African scientific leaders in NCDs.

While the GSK component will be managed as an independent laboratory, it will have full access to wider GSK R&D expertise and infrastructure. The majority of the research will be conducted in Africa by African researchers, with GSK contributing resources and expertise. Examples of the kinds of support GSK could provide for African principal investigators in this collaborative framework include clinical study design, biostatistics, genetic analysis expertise, bio-informatics, epidemiology and therapeutic expertise in cardiovascular, metabolic and respiratory medicine, and oncology.

A key aim of the Open Lab will be to support a robust R&D training programme in collaboration with leading academic groups, linked with research centres in Africa to build local scientific capability. The training programme will be integrated with the activities of the laboratory; GSK will aim to ensure the active involvement of local scientists in the research projects so that sustainable and local expertise will be built. The Open Lab will also support the education and training of African scientific researchers through partnering African researchers with GSK/academic researchers. Furthermore, the Africa NCD Open Lab will build on existing GSK partnerships and establish capability, combining the strengths of a large research-based healthcare company with academic and field experts.

An independent scientific advisory board will be established and charged to provide input on the strategy of the collaborative R&D effort, support the identification of high-quality and impactful research projects for inclusion in the portfolio, and monitor delivery progress. The scientific advisory board will be chaired by a leading African scientist, will include recognised external experts in the field, and will have majority African representation. Specific research opportunities will be reviewed and approved by the board and teams assembled by GSK.

In November 2014, the first call for research proposals was launched. Up to £4 million will be made available to fund research proposals. It is anticipated that funded projects will generate new knowledge on the unique disease mechanisms, pathophysiology and aetiology of NCDs in African patients.

## Ambition by 2025

By 2025, the aim is to initiate and deliver 25 high-impact research projects whose outputs will lead to better understanding of NCDs in Africa, and the improved use of medicines, contributing towards reaching the World Health Assembly goal to reduce avoidable mortality from NCDs by 25% by 2025. Moreover, the Open Lab will partner with and contribute towards developing NCD research capability at up to 10 African research centres. A detailed search and evaluation of the relevant academic and clinical laboratories will be conducted to identify optimal partners. These centres will be recognised as emerging worldclass centres of excellence for R&D on NCDs in Africa, and will attract and retain the next generation of scientific talent in Africa.

(Fig. 1)

**Fig. 1.**
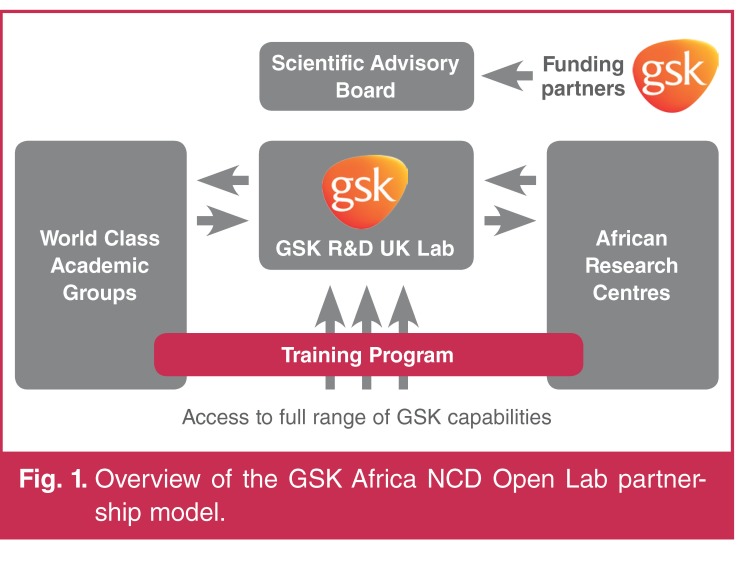
Overview of the GSK Africa NCD Open Lab partnership model.
